# Successful Isolation of Viable Adipose-Derived Stem Cells from Human Adipose Tissue Subject to Long-Term Cryopreservation: Positive Implications for Adult Stem Cell-Based Therapeutics in Patients of Advanced Age

**DOI:** 10.1155/2015/146421

**Published:** 2015-04-05

**Authors:** Sean M. Devitt, Cynthia M. Carter, Raia Dierov, Scott Weiss, Robert P. Gersch, Ivona Percec

**Affiliations:** ^1^Thomas Jefferson University Hospital, 132 S 10th Street No. 763J, Philadelphia, PA 19107, USA; ^2^Western University of Health Sciences, COMP. 309 E. Second Street, Pomona, CA 91766-1854, USA; ^3^Division of Plastic Surgery, Department of Surgery, Hospital of the University of Pennsylvania, 3400 Civic Center Boulevard, Philadelphia, PA 19104, USA; ^4^The Wistar Institute, 3601 Spruce Street, Philadelphia, PA 19104, USA

## Abstract

We examined cell isolation, viability, and growth in adipose-derived stem cells harvested from whole adipose tissue subject to different cryopreservation lengths (2–1159 days) from patients of varying ages (26–62 years). Subcutaneous abdominal adipose tissue was excised during abdominoplasties and was cryopreserved. The viability and number of adipose-derived stem cells isolated were measured after initial isolation and after 9, 18, and 28 days of growth. Data were analyzed with respect to cryopreservation duration and patient age. Significantly more viable cells were initially isolated from tissue cryopreserved <1 year than from tissue cryopreserved >2 years, irrespective of patient age. However, this difference did not persist with continued growth and there were no significant differences in cell viability or growth at subsequent time points with respect to cryopreservation duration or patient age. Mesenchymal stem cell markers were maintained in all cohorts tested throughout the duration of the study. Consequently, longer cryopreservation negatively impacts initial live adipose-derived stem cell isolation; however, this effect is neutralized with continued cell growth. Patient age does not significantly impact stem cell isolation, viability, or growth. Cryopreservation of adipose tissue is an effective long-term banking method for isolation of adipose-derived stem cells in patients of varying ages.

## 1. Introduction

Adipose-derived stem cells (ASCs) are adult mesenchymal stem cells that have garnered significant attention since their description in humans in 2001 by Zuk et al. subsequent to their initial identification in animal models [1, 2]. This is in part because the use of embryonic stem cells has been limited by multiple ethical, functional, and therapeutic dilemmas [3] and because the isolation of other adult multipotent stem cells, such as bone marrow derived stem cells, often requires complex and painful harvesting procedures resulting in low cellular yields [2]. In contrast, ASCs possess multiple characteristics that make them ideal for use in regenerative medicine applications. ASCs are multipotent, are abundant in human subcutaneous adipose tissue [4, 5], and can be harvested using minimally invasive procedures [4, 6, 7]. Significantly, the therapeutic effects of ASCs can also be garnered by the use of ASC-enriched stromal vascular fractions (SVF) without additional enzymatic isolation, a preparation consistent with good manufacturing practice (GMP), as defined by both the European Medicines Agency and the Food and Drug Administration [8].

Because ASCs can differentiate into any cell type of mesenchymal origin, including muscle, fat, bone, and cartilage, they have been hypothesized to have broad clinical applications in regenerative medicine including cellular repair after myocardial infarction, breast reconstruction, bone and cartilage regeneration after trauma, cancer, and autoimmune disorders [9]. Recent data suggest that ASCs are further able to differentiate into hepatocytes [10] and neural cells [11], extending their utility to the treatment of liver failure and brain injury, among others. In addition to their ability to differentiate and directly renew cellular populations, the benefits of ASCs further extend to their strong paracrine signaling mechanisms that confer protective effects in multiple pathological pathways, including inflammation, wound healing, neurodegeneration, and cancer [12, 13]. As the potential therapeutic applications of ASCs continue to expand, questions regarding the optimal technical management of ASCs become increasingly important to answer. Although we have increasing supplies of ASCs from the growing number of abdominoplasties and liposuction procedures performed each year [4], most current ASC investigations are performed on freshly isolated cells. These ASCs may not accurately reflect the clinical response when ASCs are isolated from cryopreserved specimens, as would be expected in future clinical scenarios with the rapid development of biobanking. Consequently, further research is required to examine the effects of tissue cryopreservation and ASC biobanking to safely and effectively optimize the therapeutic benefits of ASCs.

Several animal models have previously examined the effect of cryopreservation on ASCs. It has been shown that human lipoaspirate frozen for seven days and injected into mice displayed similar fat graft growth and resorption rates compared to freshly injected lipoaspirate [14]. Likewise, fat isolated from the inguinal region of mice that was frozen for six months demonstrated similar viability as freshly isolated tissue injected into mice [15]. A porcine model further demonstrated that isolated ASCs may be frozen for three to twelve months without inducing changes in surface markers, doubling time, and senescence markers or causing chromosomal abnormalities [16].

A limited number of studies in humans have examined the effects of cryopreservation on lipoaspirate and isolated ASCs. Lipoaspirate frozen for less than a month demonstrated no change in phenotypic markers, proliferative capacity, or differentiation potential [17]. Furthermore, cryopreserved ASCs have been shown to retain their differentiation potential and capacity when frozen for up to six months [18]. A study of almost 2500 lipoaspirates frozen for 3–6 months and subsequently used for facial rejuvenation revealed similar results in surgeon and patient satisfaction when compared to freshly injected lipoaspirate [19], though this study was not quantitative. Despite these pieces of data, there remains a paucity of studies examining the effects of long-term cryopreservation on primary human adipose tissue as a natural biobanking reservoir of ASCs. To our knowledge, the only other investigation that examined the effects of long-term (≤4 years) cryopreservation on human ASCs focused on marker profile and differentiation capabilities but not ASC proliferative ability [20]. Several studies have suggested that suboptimal cryopreservation may negatively impact ASC membrane integrity and function [21–23]. In addition, advancing patient age is believed to correlate with impaired ASC differentiation and growth profiles [24, 25]. To address these observations, we examine here whether there is a negative correlation between ASC isolation, viability, and growth in relation to increased duration of adipose tissue cryopreservation and advancing patient age.

## 2. Materials and Methods

### 2.1. Adipose Tissue Harvest

Subcutaneous abdominal fat was excised during abdominoplasties between November 2010 and January 2015 from patients of different ages. Tissue was obtained from 32 (1 male and 31 female) patients, chosen at random. All procedures were conducted using informed consent under the University of Pennsylvania IRB approval (Protocol number 812150). On the day of the procedure, tissue was excised, maintained on ice, transported to the lab, aliquoted into 50 mL conical tubes, and stored at −70°C. No cryopreservation or other agents were used in the freezing of the whole adipose tissue specimens. Tissue from 32 patients with an age range of 26–62 years (average 43.2 ± 9.7 years) and cryopreservation time (−70°C) of 2–1159 days (average 596.4 ± 369.9 days) was analyzed. Average patient BMI was 28 ± 5 kg/m^2^ and did not differ significantly between the young (27 ± 3 kg/m^2^) and advanced age groups (29 ± 6 kg/m^2^). The majority of patients were of Caucasian descent (87%), while the remaining patients were of African American descent (13%), without significant differences between the young and advanced age groups. No patients had been diagnosed as prediabetic or diabetic prior to adipose isolation.

### 2.2. Isolation of Adipose-Derived Stem Cells (ASC)

Standard methods for isolating and purifying ASCs, separating them from the stromal vascular fraction containing fibroblasts, pericytes, preadipocytes, monocytes, and macrophages, as well as smooth muscle, endothelial progenitor, and red blood cells of the SVF, have been well established and were employed here [2, 26, 27]. At defined dates, whole adipose tissue was thawed in the original 50 mL conical tubes at room temperature and stem cells were isolated from 10 g of tissue using a standard collagenase protocol [28]. Briefly, tissue was quickly washed (1xPBS/penicillin/streptomycin), minced, and digested in 15 mL 37°C warmed Digestion Media (1x Dulbecco's Modified Eagle Medium (DMEM, Gibco of Life Technologies Co., Norwalk, CT) with 0.1% collagenase (Worthington Biomedical Co, Lakewood, NJ), 1% Penicillin/Streptomycin (Corning, Christiansburg, VA), 0.008% Biotin (Sigma, Bloomington, MN), and 0.004% Pantothenate (Sigma, Bloomington, MN)) while shaking at 37°C and 180 rpm for one hour (vortexing each sample every 10 minutes). Samples were removed and 20 mL DMEM was added to each tube. Samples were then filtered using sterile funnels and gauze and spun at 800 g for ten minutes to allow for complete cell/layer-separation. The lipid layer, adipocyte layer, and media were removed carefully, leaving the Stromal Vascular Fraction pellet. Red blood cell lysis buffer (ZenBio, Durham, NC) was added to each tube and the pellet was allowed to lyse in buffer for 10 minutes at room temperature. 15 mL of DMEM was added and the tubes were spun at 800 g for 10 minutes. The supernatant was removed and 1 mL of Stem Cell Media (1xDulbecco's Modified Eagle Medium/F12 (DMEM/F12, Gibco of Life Technologies Co., Norwalk, CT) supplemented with 1% Penicillin/Streptomycin (Gibco of Life Technologies Co., Norwalk, CT) and 10% FBS (Serum Source International, Charlotte, NC)). The dissolved SVF pellet containing ASCs was transferred to a 6-well plate, after filtration through a 70 *μ*m cell strainer (FisherBrand, Pittsburgh, PA). An additional 2 mL of Stem Cell Media was rinsed through the strainer to obtain any residual cells from the filter. The plate was incubated at 37°C in 5% CO_2_ and the Stem Cell Media was changed 48 hours after plating to remove debris and nonadherent cells.

### 2.3. ASC Analysis

Cells from the SVF pellet were grown in Stem Cell Media (as described above), that was changed twice weekly. In accordance with accepted ASC isolation protocols, 48 hours after SVF pellet plating, viable and adherent cells were considered to represent adipose-derived stem cells [8, 17, 26]. 17 days after SVF pellet plating (initial ASC analysis), the number of live cells and cell viability were measured using the Countess Automated Cell Counter (Invitrogen, Carlsbad, CA) to quantify cell number and cell viability was determined by the exclusion of Trypan blue stain (Life Technologies, Norwalk, CT). At this time, representative ASC lines (*n* = 28, p0-1) were replated to a density of 1 × 10^5^ cells/well on a 12-well plate. Cells were subsequently measured again at 9, 18, and 28 days after the initial analysis. Data were analyzed with respect to the following independent variables: cryopreservation time: <1 year (*N* = 10), 1-2 years (*N* = 5), and >2 years (*N* = 17), and patient age: <40 years (*N* = 12) versus ≥40 years (*N* = 20) and <50 years (*N* = 23) versus ≥50 years (*N* = 9).

### 2.4. FACS Analysis

ASCs were grown until confluent, trypsinized, and pelleted by centrifugation at 500 g for five minutes. For fluorescence-activated cell sorting (FACS) analysis, approximately 2.5 × 10^5^ cells were resuspended in 100 *μ*L FACS buffer containing 0.1% BSA (Fisher Scientific, Philadelphia, PA) in PBS. For FACS analysis of surface markers, each sample was incubated with 5 *μ*L of anti-human PE-conjugated, PE-Cy7-conjugated, or APC-conjugated antibodies: CD34 (BD Biosciences, San Jose, CA), CD45, and CD105 (eBioscience, San Diego, CA), and then diluted in FACS buffer for 30 minutes at 4°C. Unstained cells and single-color-stained cell controls were prepared for compensation. After incubation, the excess and nonspecifically bound antibodies were removed by multiple washes in FACS buffer. Cells were pelleted and resuspended in 500 *μ*L of FACS buffer and transferred to polypropylene tubes. Propidium Iodide (Sigma, Bloomington, MN) staining was used to determine cell viability. The surface expression of live cells (approximately 10^4^ cells per patient) was characterized by FACS (Wistar Institute, Philadelphia PA) using the BD FACSCalibur flow cytometer (BD Biosciences, San Jose, CA, USA). Results were analyzed using FlowJo software version 10.0.6 (Three Star Data Analysis Software, Ashland, OR).

### 2.5. Adipogenesis Assay

ASCs were seeded in a 24-well plate at a density of 5 × 10^3^ cells/well and grown in adipogenic media comprised of DMEM/F12 Medium supplemented with 10% FBS, 1 *μ*mol/L dexamethasone, 10 *μ*g/mL insulin, and 0.5 mmol/L isobutylmethylxanthine (Sigma, Bloomington, MN). ASCs were grown for 14 days, with media changes every 3 days, after which ASCs were stained with Oil Red O (Sigma, Cloomington, MN) as per manufacturer's protocol.

### 2.6. Osteogenesis Assay

ASCs were seeded in a 24-well plate at a density of 5 × 10^3^ cells/well and grown in osteogenic media comprised of DMEM/F12 Medium supplemented with 10% FBS, 0.1 *μ*mol/L dexamethasone, 10 mmol/L *β*-glycerol phosphate, and 50 *μ*mol/L ascorbate. Cells were grown for 21 days, with media changes every 3-4 days, after which ASCs were stained with alkaline phosphatase (Amresco, Solon, OH) as per manufacturer's protocol.

### 2.7. qRT-PCR Analysis

Total mRNA was extracted from individual ASC lines (*n* = 11) and the human lung fibroblast IMR90 cell line (*n* = 3) using standard TRIzol-chloroform extraction (Invitrogen, Carlsbad, CA) and the RNeasy Mini Kit (Qiagen, Valencia, CA). cDNA was produced using a High Capacity RNA-to-cDNA Kit (Applied Biosystems of Life Technologies Co., Norwalk, CT) as per manufacturer instructions. Forward and reverse primers were designed for GAPDH (F: CGC­TCT­CTG­CTC­CTC­CTG­TT, R: GCG­ACG­CAA­AAG­AAG­ATG), Nanog (F: CTG­TGA­TTT­GTG­GGC­CTG­AA, R: CAG­AAG­ACA­TTT­GCA­AGG­ATG­G), Oct4 (F: GCC­CGA­AAG­AGA­AAG­CGA­AC, R: CAG­GTT­GCC­TCT­CAC­TCG­GT), and Sox2 (F: GCA­AGA­TGG­CCC­AGG­AGA­A, R: GCT­TGC­TGA­TCT­CCG­AGT­TGT). qPCR was prepared as a 10 *μ*L reaction using a SYBR Green PCR Master Mix Kit (Applied Biosystems of Life Technologies Co., Norwalk, CT), run on a 7900HT Fast Real-Time PCR machine (Applied Biosystems of Life Technologies Co., Norwalk, CT), and analyzed using SDS 2.4 software. Transcript levels were normalized against expression levels of the housekeeper GAPDH using the 2^−ΔΔCT^ method. Three technical replicates were performed for each assay and the results were averaged.

### 2.8. Statistical Analysis

Student *T*-test and multivariate regression analysis were performed using Microsoft Excel software (Microsoft, Redmond, WA). Unpaired, two-tailed *T*-tests were utilized to generate *P* values comparing all involved cohorts.

## 3. Results

### 3.1. ASC Isolation and Growth Relative to Cryopreservation Duration

**Figure 1:**
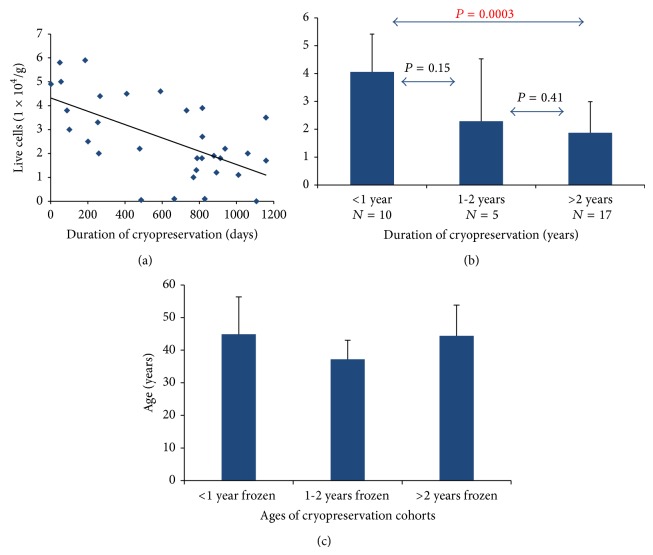
Live ASC isolation relative to duration of cryopreservation. (a) ASCs were isolated from 32 patients whose adipose tissue was cryopreserved for varying amounts of time (range 2–1159 days, average 596.4 × 10^4^ ± 369.9 × 10^4^ cells/g tissue). Live ASCs isolated ranged from 0 to 95.5 × 10^4^ cells/g adipose, average 63.8 × 10^4^ ± 30.6 × 10^4^ cells/g tissue, showing a trend toward decreased live ASC isolation with increasing ASC cryopreservation duration. (b) Live cell count was compared relative to cryopreservation duration in 3 cohort groups: <1 year (*N* = 10), 1-2 years (*N* = 5), and >2 years (*N* = 17). A significant decrease in live ASC isolation was observed between the >2 years and <1 year cryopreservation duration cohorts; *P* = 0.0003. (c) Patient age was compared for each of the cryopreservation cohort groups and no significant differences were found between any group; *P* > 0.05.

Live ASCs isolated ranged from 0 to 5.9 × 10^4^ cells/g adipose tissue harvested, averaging 2.95 × 10^4^ ± 2.5 × 10^4^ cells/g tissue. ASC isolation from frozen tissue of cohorts <1 year, 1-2 years, and >2 years had average live cell count values of 4.06 × 10^4^ ± 1.36 × 10^4^ cells/g tissue, 2.29 × 10^4^ ± 2.24 × 10^4^ cells/g tissue, and 1.87 × 10^4^ ± 1.12 × 10^4^ cells/g tissue, respectively. We observed an inverse relationship between the number of live ASCs isolated in relation to the number of days the tissue was frozen (*R* ^2^ = 0.1093, Figure 1(a)). There was a significantly greater number of live ASCs isolated from samples frozen <1 year compared to those frozen >2 years (*P* = 0.0003). No significant differences were found between other groups (Figure 1(b)). Multivariate regression analysis demonstrated no significant difference in the number of cells isolated relative to patient age or other demographic variables (*P* > 0.05). In contrast, cryopreservation length was independently associated with initial number of cells isolated irrespective of patient age (*P* < 0.001, Figure 1(c)).

**Figure 2:**
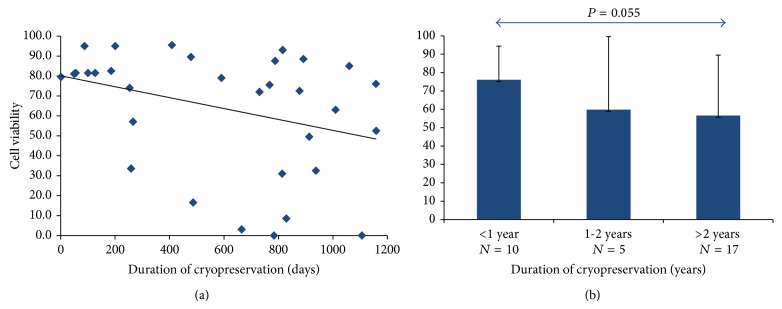
Initial ASC viability relative to cryopreservation duration. (a) A trend toward decreasing ASC viability with increasing ASC cryopreservation duration was observed. (b) ASC viability was compared relative to cryopreservation duration in 3 cohort groups: <1 year (*N* = 10), 1-2 years (*N* = 5), and >2 years (*N* = 17). No significant differences were observed between the three groups.

Tissue cohorts of cryopreservation duration of <1 year, 1-2 years, and >2 years demonstrated average viability of 76.05 ± 18.36%, 59.83 ± 39.68%, and 56.54 ± 32.99%, respectively (Figure 2). No significant differences were observed between the three groups, although a negative trend between the <1 year and >2 years cohorts was observed (*P* = 0.055, Figure 2).

**Figure 3:**
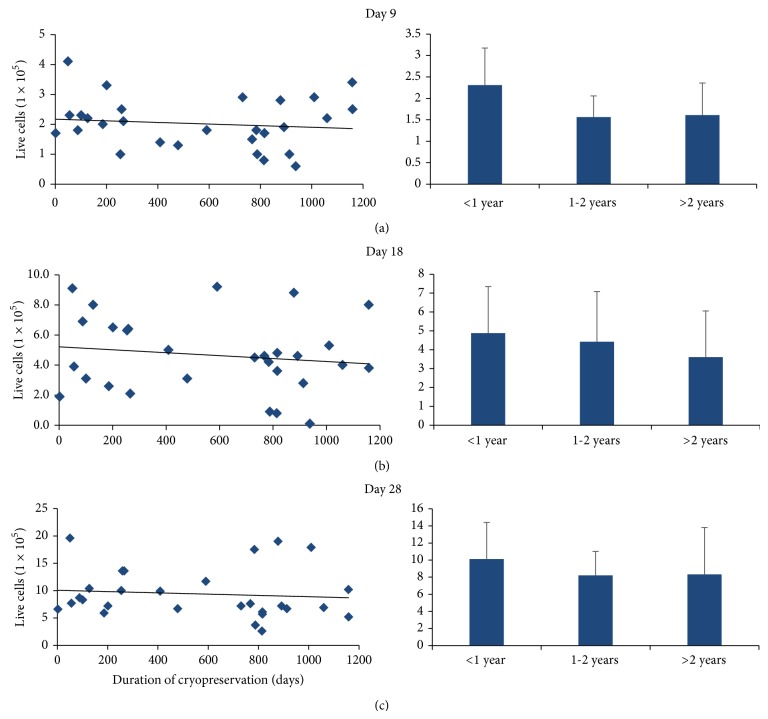
Live ASCs during extended cell growth relative to cryopreservation duration. ASCs from each patient were plated to a density of 1 × 10^5^ cells/well and counted after (a) 9, (b) 18, and (c) 28 days to characterize the effect of cryopreservation duration on continued ASC growth. We observed sustained ASC growth irrespective of cryopreservation duration. Cell counts were compared relative to cryopreservation duration in 3 cohort groups: <1 year (*N* = 10), 1-2 years (*N* = 5), and >2 years (*N* = 17), and were not found to be significantly different.

ASCs from each patient were plated to a density of 1 × 10^5^ cells/well after initial analysis and subsequently counted 9, 18, and 28 days later to examine delayed effects on cell growth secondary to cryopreservation. We observed no significant differences in ASC growth when comparing duration of cryopreservation for the <1 year, 1-2 years, and >2 years cohorts when cell number was analyzed after 9 days (2.31 × 10^5^ ± 0.86 × 10^5^ cells, 1.56 × 10^5^ ± 0.49 × 10^5^ cells, and 1.61 × 10^5^ ± 0.75 × 10^5^ cells, resp.), 18 days (4.88 × 10^5^ ± 2.47 × 10^5^ cells, 4.42 × 10^5^ ± 2.66 × 10^5^ cells, and 3.61 × 10^5^ ± 2.44 × 10^5^ cells, resp.), or 28 days in culture (10.12 × 10^5^ ± 4.27 × 10^5^ cells, 8.21 × 10^5^ ± 2.8 × 10^5^ cells, and 8.33 × 10^5^ ± 5.48 × 10^5^ cells, resp.; Figures 3(a)–3(c)).

### 3.2. ASC Isolation and Growth Relative to Patient Age

**Figure 4:**
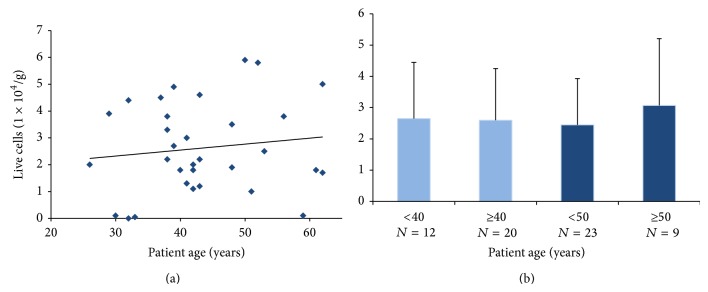
Live ASC isolation relative to patient age. (a) ASCs were isolated from 32 patients of different ages (range 26–62 years, average 43.2 ± 9.7). Live ASCs isolated ranged from 0 to 95.5 × 10^4^ cells/g adipose, average 63.8 ± 30.6, with no clear correlation between ASC isolation and patient age. (b) Live cell count was compared relative to patient age across 4 cohort groups: <40 years (*N* = 12) versus ≥40 years (*N* = 20); <50 years (*N* = 23) versus ≥50 years (*N* = 9); and <40 years (*N* = 12) versus ≥50 years (*N* = 9). No significant differences in live ASC isolation were observed between any of the age cohorts; *P* > 0.05.

Live ASCs isolated ranged from 0 to 5.9 × 10^4^ cells/g tissue, averaging 2.95 × 10^4^ ± 2.5 × 10^4^ cells/g tissue with no clear correlation between ASC isolation and patient age (Figure 4). The initial live cells were counted for each frozen tissue sample and compared between the following age cohort pairs: <40 years versus ≥40 years (2.65 × 10^4^ ± 1.8 × 10^4^ cells/g tissue and 2.6 × 10^4^ ± 1.65 × 10^4^ cells/g tissue, resp.), <50 years versus ≥50 years (2.44 × 10^4^ ± 1.48 × 10^4^ cell/g tissue and 3.07 × 10^4^ ± 2.14 × 10^4^ cells/g tissue, resp.), and <40 years versus ≥50 years (2.65 × 10^4^ ± 1.8 × 10^4^ cells/g tissue and 3.07 × 10^4^ ± 2.14 × 10^4^ cells/g tissue, resp.). No significant differences in live ASC isolation were observed between any of the age cohorts, *P* > 0.05 (Figure 4).

**Figure 5:**
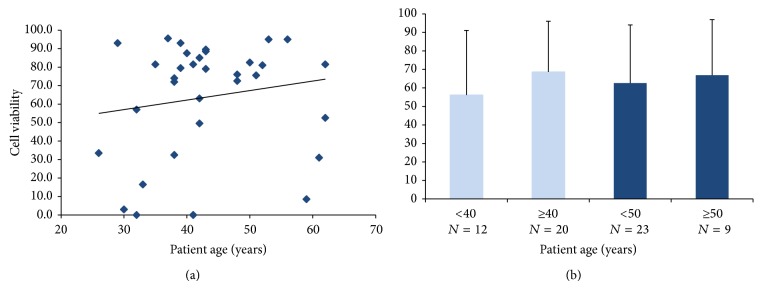
Initial ASC viability relative to patient age. (a) There was a modest trend toward increasing ASC viability with advancing patient age. (b) ASC viability was compared relative to patient age across 4 cohort groups: <40 years (*N* = 12) versus ≥40 years (*N* = 20); <50 years (*N* = 23) versus ≥50 years (*N* = 9); and <40 years (*N* = 12) versus ≥50 years (*N* = 9). No significant differences in ASC viability were observed between any of the age cohorts; *P* > 0.05.

Similarly, no significant differences in initial ASC viability relative to patient age were observed between groups, although a modest increase with advancing patient age was noted (Figure 5). ASC viability was compared between the following age cohorts: <40 years versus ≥40 years (54.13 ± 35.61% and 68.73 ± 27.29%, resp.), <50 years versus ≥50 years (61.8 ± 31.85% and 66.94 ± 30.03%, resp.), and <40 years versus ≥50 years (54.13 ± 35.61% and 66.94 ± 30.03%, resp.). No significant differences in ASC viability were observed between any of these age cohorts, *P* > 0.05 (Figure 5).

**Figure 6:**
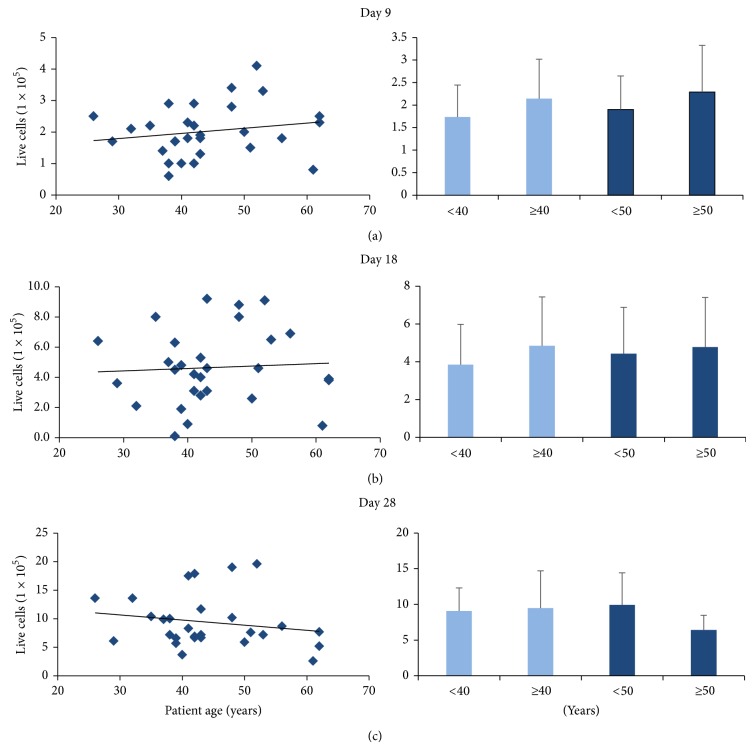
Live ASCs during extended cell growth relative to patient age. ASCs from each patient were plated to a density of 1 × 10^5^ cells/well and viability was assayed after (a) 9, (b) 18, and (c) 28 days to characterize the effect of patient age on continued ASC viability. We observed sustained ASC viability irrespective of patient age. Cell counts were compared relative to patients across 4 cohort groups: <40 years (*N* = 12) versus ≥40 years (*N* = 20); <50 years (*N* = 23) versus ≥50 years (*N* = 9); and <40 years (*N* = 12) versus ≥50 years (*N* = 9). Live cell number was not found to be significantly different in any of the comparisons.

ASCs from each patient were plated to a density of 1 × 10^5^ cells/well and counted 9, 18, and 28 days later to examine and to characterize the effect of patient age on continued ASC growth. We observed no significant differences in ASC growth relative to patient age when the average cell number was compared for the following age groups: (1) <40 years versus ≥40 year cohorts after 9 days of growth (1.73 × 10^5^ ± 0.7 × 10^5^ cells and 2.14 × 10^5^ ± 0.88 × 10^5^ cells, resp.), 18 days of growth (3.85 × 10^5^ ± 2.13 × 10^5^ cells and 4.85 × 10^5^ ± 2.59 × 10^5^ cells, resp.), or 28 days of growth (9.09 × 10^5^ ± 3.22 × 10^5^ cells and 9.49 × 10^5^ ± 5.2 × 10^5^ cells, resp.) in culture; (2) <50 years versus ≥50 year cohorts after 9 days of growth (1.9 × 10^5^ ± 0.75 × 10^5^ cells and 2.29 × 10^5^ ± 1.04 × 10^5^ cells, resp.), 18 days of growth (4.44 × 10^5^ ± 2.44 × 10^5^ cells and 4.78 × 10^5^ ± 2.63 × 10^5^ cells, resp.), or 28 days of growth (9.92 × 10^5^ ± 4.49 × 10^5^ cells and 6.41 × 10^5^ ± 2.05 × 10^5^ cells, resp.) in culture; (3) <40 years versus ≥50 cohorts after 9 days of growth (1.73 × 10^5^ ± 0.7 × 10^5^ cells and 2.29 × 10^5^ ± 1.04 × 10^5^ cells, resp.), 18 days of growth (3.85 × 10^5^ ± 2.13 × 10^5^ cells and 4.78 × 10^5^ ± 2.63 × 10^5^ cells, resp.), or 28 days of growth (9.09 × 10^5^ ± 3.22 × 10^5^ cells and 6.41 × 10^5^ ± 2.05 × 10^5^ cells, resp.; Figures 6(a)–6(c)).

### 3.3. ASC Phenotype Confirmation

**Figure 7:**
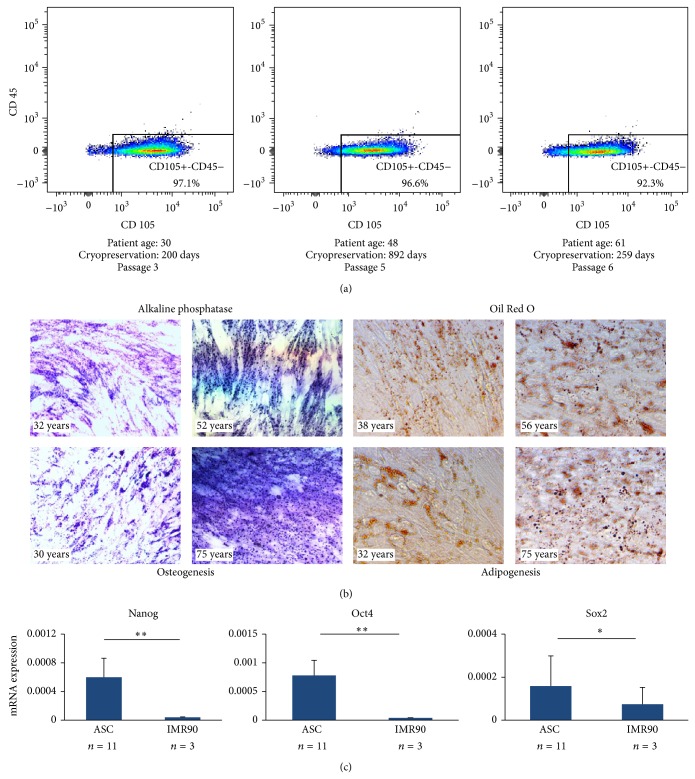
ASC phenotype confirmation. (a) ASCs were analyzed via FACS analysis for the following markers: CD105, CD34, and CD45. The data from representative patient cohorts are shown here. As predicted, the great majority of ASCs were CD105+ and CD45−, while the CD34 marker showed variable expression (data not shown), consistent with prior studies. (b) ASCs isolated from all subgroups were capable of undergoing both osteogenic and adipogenic differentiation, as demonstrated by positive staining with alkaline phosphatase and Oil Red O, respectively. (c) Pluripotency gene expression was measured via qRT-PCR for Nanog, Sox2, and Oct4 and, as expected, was found to be higher in ASCs compared to terminally differentiated IMR90 cells. ^∗^ *P* < 0.05 and ^∗∗^ *P* < 0.01.

To confirm the stem cell phenotype of ASCs isolated from cryopreserved adipose, cells from representative samples were analyzed via FACS analysis for the following markers: CD105, CD34, and CD45. CD105 served as a positive marker of human mesenchymal stem cells, CD45 as a negative marker for mesenchymal stem cells, and CD34 as a variable mesenchymal stem cell marker that is frequently lost upon extended growth in culture. As predicted, the great majority of ASCs were CD105+ and CD45− (Figure 7(a)), while the CD34 mark was variable, consistent with prior studies (data not shown). In addition, we observed ASCs isolated from all subgroups to be capable of undergoing both osteogenic and adipogenic differentiation, as demonstrated by positive staining with alkaline phosphatase and Oil Red O, respectively (Figure 7(b)). Finally, we observed the expected increased expression of pluripotency genes Nanog, Sox2, and Oct4 via qRT-PCR within representative ASC cell lines (*n* = 11) comprising a range of patient ages (26–62 years) and cryopreservation times (2–1159 days) as compared to IMR90 (*n* = 3) controls (Figure 7(c)).

## 4. Discussion

Because they are easily harvested, abundant, and able to differentiate into a wide variety of cell types, ASCs have the potential to become a powerful tool in regenerative medicine [4–7]. The use of fresh adipose tissue for ASC isolation is not feasible in many clinical or research scenarios, as therapeutic interventions and research investigations may be required months or years after tissue harvest. Unfortunately, our current understanding of available long-term biobanking/preservation methods for ASC isolation remains inadequate for conducting safe and effective ASC-based interventions. While several recent studies evaluated the efficacy of adipose tissue cryopreservation on ASCs, to date most research has examined animal models [14–17], used a short interval of cryopreservation [18, 19, 29, 30], or has been limited to ASC differentiation and marker expression analyses [20]. To address these limitations, we investigated the effects of long-term adipose tissue cryopreservation and patient age on human ASC isolation, viability, and growth.

Our findings suggest that long-term cryostorage (>2 years) significantly reduces the number of live ASCs isolated relative to short-term cryostorage (<1 year, Figure 1). Furthermore, ASCs isolated from longer preserved adipose tissue demonstrate a trend toward reduced viability (<1 year versus >2 years, *P* = 0.055, Figure 2). These findings are consistent with recent data demonstrating that stem cells grow very slowly, particularly after being thawed after cryopreservation [31, 32]. However, while fewer ASCs are isolated per gram of adipose after long-term cryopreservation, these cells maintain their robustness, perhaps suggesting that surviving ASCs may harbor a certain protective advantage. In support of this hypothesis is the observation that ASCs from different cryopreservation groups expanded throughout a 28-day time course grow at similar rates (Figures 3(a)–3(c)). Our data further demonstrate that ASCs isolated from adipose tissue that has been cryopreserved for long periods of time retain their proliferative ability while maintaining their stemness (Figures 7(a)–7(c)). Taken together, our observations suggest that differences in viability and growth observed at initial ASC isolation relative to duration of adipose tissue cryopreservation are deficits limited to early cell isolation that are neutralized upon continued ASC expansion.

Consequently, our data support whole adipose tissue cryopreservation as a valuable method for long-term ASC preservation. The observation that patient age had little impact on initial cell number, viability, or long-term cell growth further supports the suitability of this biobanking technique (Figures 4–6) and the preservation of substantial ASC counts even in fragile tissues from elderly patients that may not tolerate additional interventions without significant cellular loss. Importantly, the method of whole adipose tissue cryopreservation avoids any potential adverse effects caused by lipoaspirate-induced cellular trauma or the use of cryopreservative media, further enhancing the utility of this biobanking approach for clinical regenerative medicine applications.

This study provides evidence that whole adipose tissue cryopreservation can serve as the gold standard for long-term ASC biobanking. Because of the incremental decrease in subcutaneous adipose tissue mass with advancing patient age, it behooves us, when feasible, to bank this valuable tissue prior to its diminution, ideally by middle age. Additional studies are required to further elucidate putative effects of long-term cryopreservation on precise ASC functions and differentiation potentials in patients of different ages. This is particularly important in light of recent literature questioning the ability of directly cryopreserved ASCs to undergo osteogenic differentiation [33, 34]. Future investigations in cryopreserved whole adipose tissue will be critical for the development of human adipose tissue banks and ASC isolation for regenerative medicine applications.

## 5. Conclusions

Adipose-derived stem cells are multipotent, abundant, and easily isolated cells that represent a powerful tool for regenerative medicine. Unfortunately, much of the research regarding these cells is performed on fresh lipoaspirates that may not be readily available in therapeutic clinical scenarios. Though cryopreservation technologies continue to advance, the effects of long-term adipose tissue cryopreservation on ASC isolation have not been adequately characterized and remain a limiting factor in advancing ASC-based regenerative medicine applications. Herein we demonstrate that long-term adipose tissue cryopreservation (>2 years at −70°C) does significantly reduce the number of ASCs initially isolated per gram of tissue. However, this primary attenuation in ASC isolation is neutralized upon extended culturing as demonstrated by comparable ASC proliferative capacity, irrespective of cryopreservation duration or patient age. Together, our data suggest that ASCs are capable of enduring long-term cryopreservation while maintaining their stemness, further reinforcing their important contribution to regenerative medicine applications. We anticipate that data such as those reported here will be critical for the development of safe and effective long-term human adipose tissue biobanking technologies, optimization of ASC isolation protocols using minimal tissue/cell processing, and validation of specific clinical therapeutic applications for ASCs. In conclusion, we advocate for whole adipose tissue cryopreservation in preparation for future ASC-based regenerative medicine therapies.
